# Major maternal related determinants of non-breastfeeding among mothers in Ethiopia: A multilevel analysis from DHS Ethiopia 2016

**DOI:** 10.1371/journal.pone.0286662

**Published:** 2023-06-08

**Authors:** Amare Wondim, Masresha Asmare Techane, Chalachew Adugna Wubneh, Nega Tezera Assimamaw, Getaneh Mulualem Belay, Tadesse Tarik Tamir, Addis Bilal Muhye, Destaye Guadie Kassie, Bewuketu Terefe, Bethelihem Tigabu Tarekegn, Mohammed Seid Ali, Beletech Fentie, Almaz Tefera Gonete, Berhan Tekeba, Selam Fisiha Kassa, Bogale Kassahun Desta, Amare Demsie Ayele, Melkamu Tilahun Dessie, Kendalem Asmare Atalell, Tewodros Getaneh Alemu

**Affiliations:** 1 Department of Pediatrics and Child Health Nursing, School of Nursing, College of Medicine and Health Sciences, Specialized Hospital, University of Gondar, Gondar, Ethiopia; 2 Department of Community Health Nursing, School of Nursing, College of Medicine and Health Sciences, University of Gondar, Gondar, Ethiopia; Madda Walabu University, ETHIOPIA

## Abstract

**Introduction:**

In Ethiopia, the burden of non-breastfeeding is still high despite substantial improvements in breastfeeding. However, the determinants of non-breastfeeding were poorly understood. Therefore, the aim of this study was to identify the maternal -related factors associated with non-breastfeeding.

**Methods:**

An in-depth analysis of data from the Ethiopian Demographic and Health Survey 2016 (EDHS 2016) was used. A total weighted sample of 11,007 children was included in the analysis. Multilevel logistic regression models were fitted to identify factors associated with non-breastfeeding. A p-value < of 0.05 was used to identify factors significantly associated with non-breastfeeding.

**Results:**

The prevalence of non-breastfeeding in Ethiopia was 5.28%. The odds of not breastfeeding were 1.5 times higher among women aged 35to 49 years (AOR = 1.5 CI: 1.034, 2.267) than among women aged 15to 24 years. The odds of not breastfeeding were higher among children whose mothers had BMIs of 18.5–24.9 (AOR = 1.6 CI: 1.097, 2.368) and 25–29.9 (AOR = 2.445 CI: 1.36, 4.394) than among women with BMIs of < 18.5. In addition, not breastfeeding was also significantly associated with ANC follow-up, where mothers who had 1–3 ANC follow-up had a 54% decreased odds (AOR = 0.651 CI: 0.46,0.921) compared to mothers who had no ANC follow-up. Demographically, mothers from Somalia region were five times (AOR = 5.485 CI: 1.654, 18.183) and mothers from SNNP region were almost four times (AOR = 3.997 CI: 1.352, 11.809) more likely to not breastfeed than mothers residing in Addis Ababa.

**Conclusions:**

Although breastfeeding practices are gradually improving in Ethiopia, the number of children not breastfed remains high. Individual-level characteristics (women’s age, body mass index, and ANC follow-up) and community-level characteristics (geographic region) were statistically significant determinants of non-breastfeeding. Therefore, it is good for the federal minister of Health, planners, policy and decision- makers, and other concerned child health programmers to prioritize both individual and community factors.

## Introduction

Breastfeeding supplies nutrients for growth and development as well as immunity to infectious illnesses. The World Health Organization (WHO) has advised that every child should be breastfed exclusively for the first six months of life, with partial breastfeeding lasting until age two [1]. Despite this widely accepted advice and the advantages to both short- and long-term health, a significant number of children are still not breastfed [2,3].

Worldwide, failure to breastfeed according to guidelines and use of breast milk substitutes result in more than 800,000 infant deaths and cognitive impairments [4]. Non-breastfeeding increases the risk of obesity, type 1 and type 2 diabetes, leukemia, and sudden infant death syndrome in children [5]. Additionally, not breastfeeding is strongly linked to increased rates of mortality from infectious diseases and respiratory system infections leading to hospital admissions [6,7]. The incidence of infectious morbidity and infections such as diarrhea and pneumonia is high [4]. Furthermore, not breastfeeding presents serious difficulties for infants. For instance, infants who were not breastfed had a 3.6 and 2.4-fold higher risk of developing necrotizing enterocolitis and lower respiratory tract infections, respectively, compared to infants who were exclusively breastfed [8].

Globally, it is estimated that not breastfeeding causes yearly economic losses of around $302 billion due to the financial burden and cognitive deficits it causes. More specifically, there is a cost of $14.4 billion in premature mortality and $733.7 million in direct care expenses when infants are not optimally breastfed (6 months exclusively, one year, or longer) [5]. In addition, there are significant and high expenses to the health care system related to the morbidity and environmental costs of breast milk substitutes [2].

The problem of not breastfeeding impacts mothers as well as infants. Breast cancer, ovarian cancer, retained pregnancy weight gain, type 2 diabetes, myocardial infarction, and metabolic syndrome are all more common in non-breastfeeding mothers [5]. In addition, not breastfeeding increases the risk of postpartum hemorrhage and depression, as well as premature mortality from various diseases later in life [4].

The literature reports a number of causes for non-breastfeeding in different communities in Ethiopia. Cultural, social, and economic reasons have been mentioned as obstacles to not breastfeeding [4,9]. Access to services, cultural barriers (breastfeeding is not the norm in many communities, and it is embarrassing to do so in public), a lack of knowledge about breastfeeding, inaccurate information, the workplace environment, a lack of family and social support, breastfeeding problems, returning to work and access to supportive childcare, policies and practices of some health services and health care providers, and the promotion and marketing of infant formula were some of the specific factor that were identified [10–13].

Although UNICEF and WHO have jointly identified breastfeeding as a crucial step towards reaching SDGs 2, 3, 4, and 5, breastfeeding practices and their effects on infant survival and health are an undeniable cause for concern worldwide [14–16], a significant percentage of newborns continue not to be breastfed. Despite this fact, there is no comprehensive understanding of the current factors underlying non-breastfeeding among a nationally representative sample in Ethiopia. Therefore, this study aimed to determine maternal related factors that predict non-breastfeeding among mothers using the Ethiopian Demographic and Health Survey 2016 (EDHS).

## Methods

### Study design and setting

A cross-sectional, population-based research, the factors that contribute to non-breastfeeding have been studied. It is based on data from the 2016 Ethiopian Demographic Health Survey (EDHS) surveys. The Demographic Health Survey (DHS), a multi-round study, assesses population health with a focus on maternal and child health as well as health indicators of worldwide importance. Ethiopia is the second most populous country in Africa, with an estimated 115 million people and a total land size of 1.04 million square kilometers. The nation has a variety of geographical characteristics, with heights varying from 125 meters below sea level in the Afar Depression to 4550 meters above sea level in Ras Dejen in the Semien Mountains in the Amhara region. Ethiopia is divided into two city administrations (Addis Ababa and Dire Dawa) and twelve administrative regions (Tigray, Afar, Amhara, Oromia, SNNP, Benshangulgumuz, Somali, Gambela, Harari, Sidama, South West Ethiopia and Southern Ethiopia) (first level). The country is also divided into zones (second level), districts (third level) and kebeles (fourth level). Ethiopia has a three-tier health care system consisting of (1) primary care: consisting of health posts, health centers, and primary hospitals; (2) secondary care: consisting of general hospitals; and (3) tertiary care: consisting of specialized hospitals [17]. Due to inadequate transportation infrastructure, more than half of the population in Ethiopia lives more than 10 kilometers from the closest health care facility [18].

### Data sources, sampling technique, and study population

Data were compiled from various sources; for this study, data were taken from the 2016 EDHS for the primary outcome (i.e. non-breastfeeding status), the individual and the community factor. The EDHS surveys collect data every five years that are nationally representative. The fourth in a series of demographic and health surveys in Ethiopia is the EDHS 2016. A two-stage stratified cluster sampling technique was used to collect the data from the EDHS study. In the first part, 645 survey areas (clusters) were selected and stratified into urban and rural areas. The second step entailed selecting the homes within each of the finalized clusters. The weighted sample included 11,007 eligible children under the age of five.

#### Study variables

We used non-breastfeeding as the outcome variable (Children who never had breast feed) using EDHS 2016, from the duration of the breastfeeding section.

Factors at the individual and community levels were included as covariates. Individual factors included sociodemographic characteristics such as the highest educational level of the mother (defined as "no education," "primary," or "secondary or above") and the highest educational level of the partner ("no education," "primary," or "secondary or above"). Marital status ("never married," "currently married," "formerly married"), mother’s age ("15–24 years," "25–34 years," "35–49 years"), and the mother’s media exposure ("no" or "yes")

EDHS 2016, reported a wealth index combined by five classification: “poorest, poorer, middle, richer and richest”. In this study, wealth indexes were classified into three clusters, grouping the poorest and poorer into “poor”, richer and the richest into “rich”. Hence we had poor, middle and rich classification.

Maternal- related factor included the number of antenatal clinic visits (categorized as “0 visits”, “1–3 visits”, “4 or more visits”), place of delivery (“home” or “health facility”), and mode of delivery (“cesarean section” or “vaginal”) and maternal body mass index (BMI) measured as weight (kg)/height (m^2^). Community-level factors included place of residence (“rural” or “urban”) and geographic region.

Geographical regions were divided into nine regional states of Ethiopia; namely Afar, Amhara, Benishangul-Gumuz, Gambella, Harari, Oromia, Somali, Southern Nations Nationalities, and Peoples’ Region (SNNP), and Tigray, as well as two city administrations, Addis Ababa and Dire Dawa. Community-level indicators of women’s literacy, media exposure, and poverty level were developed by combining individual-level characteristics in order to conceptualize their impact on non- breast feeding. In order to generate the aggregate community-level predictor variables, the individual-level values from each cluster were combined, and the binary classification of the aggregate variables (high or low) was based on the distribution of the proportion values computed for each cluster. For community poverty status: by aggregating of individual maternal poverty using proportion and swilk proportion to check the normality assumption if normality assumption fail then we use median as a measure of summary statistics and for community woman literacy: aggregating of individual maternal educational level (no education, primary and secondary and above) into two cluster (poor, good) and community media exposure using individual maternal exposure status (no, yes) and considering the proportion of mother having no media exposure and swilk proportion to check the normality assumption if normality assumption fail then we use median as a measure of summary statistics. For non-normally distributed aggregate predictor variables at the community level, the median served as the cutoff number for classification. The histogram was used to ascertain whether the distribution was normal or not.

### Statistical analysis

Descriptive statistics were used to show how the background characteristics were distributed. Because the survey findings were nested and varied by cluster (census tracts). Sampling weights were used to account for the uneven selection probability between clusters and reestablish the survey’s representativeness. In this research, a mixed-effects logistic regression model was used to determine the actual relationship between not breastfeeding and different characteristics. Multilevel logistic regression models were developed to address individual and community variables related to not breastfeeding. The first of the four models was Model I (null model), which was fitted without explanatory factors to check for random variability in the intercept and to determine the intra- class correlation coefficient (ICC). Model II examined the impact of individual characteristics on outcomes. Model III focused on community-level variables. While Model IV concurrently examined both individual and community-level characteristics. In order to take statistical significance for measures of association into consideration, adjusted odds ratios with 95% confidence intervals were used (fixed effects). For measures of variation (random effects), ICC, median odds ratio (MOR), and proportional change in variance (PCV) statistics were calculated. Model comparison was based on the deviance information criteria (DIC). The model with the lowest information criterion was considered the best-fitting model.

### Ethical considerations

We requested the data from the MEASURE DHS program, and permission to download was granted. The downloaded data were used for this study only. The data set was not shared with other researchers without the consent of EDHS. All data from EDHS were kept confidential without identifying any household or individual respondent. ​In addition, as we used secondary data (national survey), informed consent was not taken.

## Results

### Individual-level characteristics of study participants

The prevalence of non-breastfeeding in Ethiopia was 5.28% (n = 581) from the overall weighted population of 11,007 children. About 52.99% of the mothers, or about half, were in the 25–34 age group. In terms of educational attainment, 66.07 and 48.52% of mothers and their husbands/partners, respectively, did not have any formal education. The majority of mothers (94.9%) were married. With a low rate of caesarean births (1.93%), 27.39% of births took place in health care facility. About 74.04% of mothers had a body mass index between 18.5 and 24.9 kg/m^2^, and 31.88% of mothers stated they had visited an antenatal facility more than four times while they were pregnant. For 66.92% of respondents, there was no media coverage (see Table 1).

**Table 1:** Background characteristics of individualS among mothers in EDHS 2016 (weighted n = 11,007).

Variables	Categories	Weighted Frequency	percent
Age of the mother	15–24	2437.61	22.14
	25–34	5833.11	52.99
	35–49	2737.06	24.86
marital status	Never married	56.93	0.52
	Currently married	10,450.74	94.9
	Formerly married	500.12	4.54
Mother education level	No education	7272.77	66.07
	Primary	2948.07	26.78
	Secondary and above	786.95	7.15
husband/partner’s educational level	No education	5,070.34	48.52
	Primary	4,113.78	39.36
	Secondary and above	1,266.62	12.12
Wealth index	Poor	5152.74	46.81
	Middle	2276.36	20.68
	Rich	3578.67	32.51
Media	No	7366.26	66.92
	Yes	3641.52	33.08
BMI	<18.5	2102.54	19.62
	18.5–24.9	7932.79	74.04
	25–29.9	542.14	5.06
	> 30	136.67	1.28
Place of delivery	Home	7992.5	72.61
	Health facility	3015.28	27.39
delivery by cesarean section	Non cesarean	10794.97	98.07
	Cesarean section	212.83	1.93
Number of ANC visits	No visit	2818.27	37.21
	1–3 visit	2341.95	30.92
	4 and above visit	2414.64	31.88

### Community-level characteristics of study participants

About 89.01% of the children resided in rural areas, with a particularly high number (44.04%) in the Oromia regions. Regarding community poverty status, 63.94% had a lower level, whereas 56.55% had more media exposure (Table 2).

**Table 2:** Background characteristics of communities among mothers in EDHS 2016 (weighted n = 11007).

Variables	Categories	Weighted frequency	Percent
Region	Tigray	710.67	6.46
	Afar	114.03	1.04
	Amhara	2070.19	18.81
	Oromia	4848.14	44.04
	Somali	506.94	4.61
	Benishangul	121.64	1.11
	SNNP	2292.67	20.83
	Gambela	26.85	0.24
	Harari	25.79	0.23
	Addis Ababa	243.94	2.22
	Dire Dawa	46.92	0.43
Residence	Urban	1210.19	10.99
	Rural	9797.58	89.01
community level poverty	Lower	7038.67	63.94
	higher	3969.11	36.06
community level woman literacy	Lower	2085.79	18.95
	higher	8921.99	81.05
community level media exposure	Lower media exposure	4783.08	43.45
	Higher exposure	6224.71	56.55

Measures of variation (random-effects) and model fit statistics.

Based on the null model, there was statistically significant variation in non-breastfeeding across communities.

About 23.56% of the variation in the odds of non-breastfeeding is attributed to community-level factors (ICC = 23.56%). After adjusting the model for individual-level factors (Model II), about 4.06% of the variation in the odds of non-breastfeeding was attributed to individual-level factors (PCV = 4.06%), while 24.3% of the variance in non-breastfeeding was explained by community-level factors (ICC = 24.3%). Based on Model III, which was adjusted for community-level factors, it was found that 22.13% of the variability among clusters was explained by community-level factors (PCV = 22.13%), and 19.37% was explained by community-level factors (ICC = 19.37%). Model IV is the best-fit model which incorporates both individual and community-level factors simultaneously. Based on this final model, about 21.74 percent of the variance in non-breastfeeding odds among communities was explained by community-level factors (ICC = 21.74%), while 9.96% of the variance in the odds of non-breastfeeding across communities (PCV = 9.96%) was explained by both individual and community-level factors. Incorporating both individual- and community-level factors, the unexplained heterogeneity in non-breastfeeding between communities was reduced from MOR of 2.6 to MOR of 2.48. (Table 3). A small number of DIC is in Model IV, indicating that the explanatory value of the model increases for Model IV. In other words, Model IV explained the determinants better than Models II and III; this makes the final model the best-fitted model than others (Table 3).

**Table 3:** Measures of variation (random intercept models) and model fit statistics in non-breast feeding in EDHS 2016.

Parameter	Null model	Model II	Model III	Model IV
ICC (%)	23.56	24.3	19.37	21.74
MOR	2.6(2.27,3.05)	2.65(2.18,3.39)	2.33(2.04,2.72)	2.48(2.05,3.16)
PCV (%)	Reference	4.06%	22.13%	9.96%
Model comparison				
Log likelihood	-2163.4248	-1089.8738	-2143.0982	-1080.4471
Deviance	4326.8496	2179.7476	4286.1964	2160.8942

### Multilevel logistic regression analysis

Maternal age, maternal education, place and type of delivery, body mass index, the media, ANC follow-up, wealth, and the community factors of residence and region were all significant in the bivariable logistic regression with p-values 0.25. Only maternal age, BMI, ANC visit, and region were associated with non-breastfeeding in the multivariable logistic regression at a p-value of 0.05. Non-breastfeeding is 1.5 times more likely among 35–49 years old women (AOR = 1.5, CI: 1.034, 2.267) than among 15–24 years- old women. The odd of non-breastfeeding in children whose mothers had a BMI 18.5–24.9(AOR = 1.6 CI: 1.097, 2.368) and 25–29.9(AOR = 2.445 CI: 1.36, 4.394) higher than those of women having BMI <18.5.

Non-breastfeeding was also significantly associated with ANC follow-up, where mothers with 1–3 ANC follow-up had a 35% decreased odds of (AOR = 0.651 CI: 0.46,0.921) compared to mothers without ANC follow- up. Demographically, the odds of non-breastfeeding five times (AOR = 5.485 CI: 1.654, 18.183) in Somali and nearly four times (AOR = 3.997 CI: 1.352, 11.809) in SNNP higher than compared to mothers residing in Addis Abeba (Table 4).

**Table 4:** Predictor of non- breastfeeding among mothers children in Ethiopia, 2016.

Variables	Categories	breast feeding status		Odds ratio			
		NBF^a^	BF^b^	COR	Model II AOR (95% CI)	Model III AOR (95% CI)	Model IV AOR (95% CI)
Age of the mother	15–24	134	2435	1	1		1
	25–34	279	5230	0.86(0.685, 1.083)	0.864(0.607, 1.232)		0.86(0.604, 1.229)
	35–49	162	2382	1.16(0.9, 1.506)	1.497(1.013, 2.212)		**1.53(1.034,2.267)** *
Mother education level	No education	382	6443	1	1		1
	Primary	121	2553	0.73(0.593, 0.923)	0.881(0.627, 1.236)		0.68(0.618, 1.22)
	Secondary and above	72	1051	1.13(0.804, 1.593)	1.114(0.653, 1.899)		1.08(0.623, 1.874)
Wealth index	Poor	302	5468	1	1		1
	Middle	82	1379	1.32(1.038, 1.702)	1.152(0.79, 1.679)		1.19(0.814, 1.742)
	Rich	191	3200	1.41(1.118,1.799)	1.351(0.938,1.944)		1.36(0.937,1.978)
Media	No	386	6765	1	1		1
	Yes	189	3282	1.28(1.055,1.567)	1.342(0.975,1.847)		1.37(0.999,1.899)
BMI	<18.5	134	2333	1	1		1
	18.5–24.9	364	6434	1.23(.967,1.576)	1.614(1.100,2.366)		**1.61(1.097,2.368)** *
	25–29.9	45	692	1.54(1.011,2.352)	2.32(1.421,4.512)		**2.44(1.36,4.394)** *
	> 30	11	214	0.97(0.434,2.203)	1.344(0.468,3.86)		1.238(0.428,3.585)
Place of delivery	Home	409	6736	1	1		1
	Health facility	166	3311	0.79(0.637,0.996)	0.801(0.565,1.136)		0.795(0.556,1.136)
delivery by caesarean section	Non caesarean	558	9759	1	1		1
	Caesarean section	17	288	1.69(1.037, 2.785)	1.782(0.922, 3.443)		1.904(0.977,3.711)
ANC visit	No visit	122	2359	1	1		1
	1–3 visit	66	2026	0.60(0.438, 0.846)	0.645(0.456, 0.911)		**0.65(0.46, 0.921)** *
	4 and above visit	94	2507	0.80(0.59,1.099)	0.832(0.587,1.179)		0.85(0.6,1.217)
community level poverty	Lower	283	5056	1		1	1
	higher	292	4991	0.888(0.744, 1.059)		1.01(0.706,1.473)	1.34(0.797,2.273)
community level woman literacy	Lower	139	2482	1		1	1
	higher	436	7565	0.83(.679, 1.02)		1.091(0.736,1.617)	1.05(0.607,1.833)
community level media exposure	Lower media exposure	290	5022	1		1	
	Higher exposure	285	5025	1.23(1.044,1.471)		1.13(0.806,1.594)	1.30(0.805,2.101)
Region	Tigray	32	993	0.54(0.238,1.236)		0.86(0.361,2.076)	2.52(0.784,8.158)
	Afar	46	1014	0.68(0.205,2.294)		1.10(0.318,3.807)	3.48(0.658,18.499)
	Amhara	26	950	0.46(0.22,0.967)		0.74(0.333,1.649)	1.69(0.562,5.108)
	Oromia	79	1501	0.90(0.452,1.815)		1.51(0.698,3.292)	2.68(0.913,7.704)
	Somali	112	1390	1.43(0.66,3.12)		2.28(0.993,5.239)	**5.48(1.654,18.183)** *
	Benishangul	54	825	1.08(0.377,3.1)		1.83(0.608,5.554)	3.48(0.693,17.49)
	SNNPR	108	1167	1.49(0.742,3.02)		2.52(1.153,5.508)	**3.99(1.352,11.809)** *
	Gambela	47	666	1.26(0.234,6.881)		1.77(0.323,9.733)	5.05(0.573,44.588)
	Harari	19	585	0.50(0.046,5.575)		0.72(0.066,7.998)	1.14(0.028,46.859)
	Addis Abeba	27	434	1		1	1
	Dire Dawa	25	522	0.91(0.201,4.195)		1.20(0.264,5.478)	2.27(0.266,19.409)
Residence	Urban	117	1852	1		1	1
	Rural	458	8195	0.68(0.476, 0.995)		0.55(0.372, 0.839)	0.65(0.363,1.169)

Notes: * p < 0.05; 1 = Reference category.

^a^ NBF: Non-breastfeeding.

^b^ BF: Breastfeeding.

## Discussion

This research aimed to assess the prevalence and determinants (individual and community-level) of non-breastfeeding in Ethiopia using nationally representative EDHS 2016 data.

According to WHO guidelines, every infant should get exclusive breast milk for the first six months of life, with partial breastfeeding continuing until age two [1]. In this demographic survey, 5.41% of children were non-breastfed. This is lower than studies conducted in Saudi Arabia, 19.2% [19], 18% in EDO state, Nigeria [20] and 19% in India [6]. Different socioeconomic and cultural norms may be the cause of the discrepancies. This finding is contrary to the principles and recommendations of the World Alliance for Breastfeeding Action, as well as the World Health Organization’s belief that more than 820,000 children could be saved annually if all infants and young children aged 0 to 23 months received the recommended amount of breast milk [1,21].

According to this research, there is an important association between the mother’s age and the children’s non-breastfed status. Compared to women aged 15–24, women aged 35–49 had greater odds of not breastfeeding. Study results from rural populations in Saudi Arabia, Nigeria, and Italy support this finding [19,20,22]. This could be because mothers feel their own amounts of breast milk are insufficient as their children get bigger. Mothers who are 35 years old or older might therefore require extra care. There is a substantial metabolic burden on breastfeeding mothers because it takes 500 kcal per day to make milk for a baby who is exclusively breastfed [5]. The current research also found an association between body mass index and not breastfeeding, with the likelihood of not breastfeeding being greater in children whose mothers had BMIs of 18.5–24.9 and 25–29.9 kg/m^2^ compared to women with BMIs under 18.5 kg/m^2^. The finding is in line with a quantitative review of the literature in Japan [23] and other studies [24,25]. This could occur as a result of overweight mothers having huge, heavy breasts that could biologically hinder a baby’s ability to suck or overweight women having a physiological pattern that negatively affects "the maternal-fetal connection [26–28]. The reason is also perhaps associated with socio-cultural factors.

According to studies, breastfeeding instruction and counselling from parents during pregnancy significantly affects mothers’ rates of early breastfeeding initiation and exclusive breastfeeding continuation [29,30]. Similar to the previous research, the current study also found a link between ANC visits and not breastfeeding, with mothers who had one to three ANC follow-up visits experiencing a 54% decrease in the likelihood of not breastfeeding as compared to mothers who did not receive ANC follow-up. This may be due to the fact that mothers will learn about the therapeutic advantages of breastfeeding during their ANC follow-up appointments, which is the main component of the service package. Additionally, it’s possible that they’ll learn about breastfeeding challenges and the consequences of infant feeding on health.

The prevalence of mothers who don’t breastfeed differs significantly across the regional states of Ethiopia. Mothers from SNNP and Somali were much less likely to choose not to breastfeed their children compared to mothers from Addis Ababa. This might be the result of the mother living in SNNP and Somalia were unaware of the potential benefit. Additionally, these areas are border regions and might make it difficult for them to use and access healthcare services. Additionally, people in these regions might be less educated and live more remote from healthcare facilities [31].

### Strengths and limitations of the study

Multilevel analysis was used to manage the hierarchical nature of the DHS data, producing accurate estimation standard errors based on weighted, nationally representative data with a large sample size. The research may also provide policymakers and programme planners with helpful information for developing efficient interventions at both the national and regional levels. This research does have some limitations due to the DHS survey’s reliance on respondents’ reports, which may be biased by recall. Drawing the cause and effect relationship is challenging due to the cross-sectional nature of the research. Because the data was gathered in 2016, it’s possible that it doesn’t accurately reflect the situation currently.

## Conclusion

Despite a slight improvement in breastfeeding practices, the number of not breasted children in Ethiopia is still significant. Age, BMI, and ANC follow-up were variables at the individual and geographic area from community levels were statistically significant predictors of not breastfeeding. Therefore, it would be advantageous if the Federal Ministry of Health, planners, policy-makers, and other child health programmers made addressing both the variables at the individual and community levels a priority.

## Abbreviations

AOR: Adjusted odds ratio
ANC: Antenatal care
BMI: Body mass index
CI: Confidence Interval
COR: Crude odds ratio
DHS: Demographic health survey
DIC: Deviance Information Criteria
EDHS: Ethiopian demographic and health survey
ICC: Intra class Correlation Coefficient
MOR: Median odds ratio
PCV: proportional change in variance
SNNP: Southern nation’s nationalities and peoples
UNICEF: United Nations international children’s fund
WHO: World Health Organization

## References

[1] WHO. Breastfeeding. 2021.https://www.who.int/health-topics/breastfeeding.

[2] Persson LÅ. Breastfeeding in low-resource settings: Not a “small matter”. Public Library of Science San Francisco, CA USA; 2018. p. e1002646. 10.1371/journal.pmed.1002646 PMC6112620 30153251

[3] Mercier RJ. Identifying risk factors for not breastfeeding: the interaction of race and economic factors: A case for seeking a local perspective. Breastfeeding Medicine. 2018;13(8):544–8. doi: 10.1089/bfm.2018.0118 30335490

[4] Walters DD, Phan LT, Mathisen R. The cost of not breastfeeding: global results from a new tool. Health policy and planning. 2019;34(6):407–17. doi: 10.1093/heapol/czz050 31236559 PMC6735804

[5] Stuebe A. The risks of not breastfeeding for mothers and infants. Reviews in obstetrics and gynecology. 2009;2(4):222. 20111658 PMC2812877

[6] Payne S, Quigley MA. Breastfeeding and infant hospitalisation: analysis of the UK 2010 Infant Feeding Survey. Maternal & child nutrition. 2017;13(1):e12263. doi: 10.1111/mcn.12263 27010760 PMC6865925

[7] Sankar MJ, Sinha B, Chowdhury R, Bhandari N, Taneja S, Martines J, et al. Optimal breastfeeding practices and infant and child mortality: a systematic review and meta‐analysis. Acta paediatrica. 2015;104:3–13. doi: 10.1111/apa.13147 26249674

[8] Chen A, Rogan WJ. Breastfeeding and the risk of postneonatal death in the United States. Pediatrics. 2004;113(5):e435–e9. doi: 10.1542/peds.113.5.e435 15121986

[9] Kong SK, Lee DT. Factors influencing decision to breastfeed. Journal of advanced nursing. 2004;46(4):369–79. doi: 10.1111/j.1365-2648.2004.03003.x 15117348

[10] Tasmania BC. Barriers to breastfeeding. 2015.http://www.breastfeedingtas.org/about/barriers_to_breastfeeding.

[11] UNICEF U, Initiative BF. Removing the barriers to breastfeeding: A call to action. Available at: Barriers to Breastfeeding Briefing Document (unicef. org. uk …; 2017.

[12] Anstey EH, Coulter M, Jevitt CM, Perrin KM, Dabrow S, Klasko-Foster LB, et al. Lactation consultants’ perceived barriers to providing professional breastfeeding support. Journal of Human Lactation. 2018;34(1):51–67. doi: 10.1177/0890334417726305 28820951

[13] Brown A. Breastfeeding as a public health responsibility: a review of the evidence. Journal of Human Nutrition and Dietetics. 2017;30(6):759–70. doi: 10.1111/jhn.12496 28744924

[14] Chandhiok N, Singh L, Singh KJ, Sahu D, Pandey A. Does breastfeeding have an effect on infant mortality in India? An analysis of national family health survey data. Open Journal of Preventive Medicine. 2015;5(09):359.

[15] Ekholuenetale M, Barrow A. What does early initiation and duration of breastfeeding have to do with childhood mortality? Analysis of pooled population-based data in 35 sub-Saharan African countries. International Breastfeeding Journal. 2021;16(1):1–9. 34876163 10.1186/s13006-021-00440-x PMC8650286

[16] Action WAfB. Breastfeeding: A Key to Sustainable Development UNICEF and WHO joint message for World Breastfeeding Week 2016. 2016.

[17] Workie NW, Ramana GN. The health extension program in Ethiopia. 2013.

[18] Ethiopia Ababa A. Abstract available from: https://wfphaconfexcom/wfpha/2012/webprogram/Paper10587html.2013.2013

[19] Azzeh FS, Alazzeh AY, Hijazi HH, Wazzan HY, Jawharji MT, Jazar AS, et al. Factors associated with not breastfeeding and delaying the early initiation of breastfeeding in Mecca Region, Saudi Arabia. Children. 2018;5(1):8. doi: 10.3390/children5010008 29301353 PMC5789290

[20] Salami LI. Factors influencing breastfeeding practices in Edo state, Nigeria. African journal of food, agriculture, nutrition and development. 2006;6(2).

[21] Action WAfB. World Breastfeeding Week–‘Breastfeeding: A key to Sustainable Development. 2021.

[22] Colombo L, Crippa BL, Consonni D, Bettinelli ME, Agosti V, Mangino G, et al. Breastfeeding determinants in healthy term newborns. Nutrients. 2018;10(1):48. doi: 10.3390/nu10010048 29304013 PMC5793276

[23] Nomura K, Minamizono S, Nagashima K, Ono M, Kitano N. Maternal Body Mass Index and Breastfeeding Non-Initiation and Cessation: A Quantitative Review of the Literature. Nutrients. 2020;12(9):2684. doi: 10.3390/nu12092684 32887461 PMC7551008

[24] Babendure JB, Reifsnider E, Mendias E, Moramarco MW, Davila YR. Reduced breastfeeding rates among obese mothers: a review of contributing factors, clinical considerations and future directions. International breastfeeding journal. 2015;10(1):1–11. 26140049 10.1186/s13006-015-0046-5 PMC4488037

[25] Ballesta-Castillejos A, Gomez-Salgado J, Rodriguez-Almagro J, Ortiz-Esquinas I, Hernandez-Martinez A. Relationship between maternal body mass index with the onset of breastfeeding and its associated problems: an online survey. International breastfeeding journal. 2020;15(1):1–13. doi: 10.1186/s13006-020-00298-5 32539791 PMC7296910

[26] Gubler T, Krähenmann F, Roos M, Zimmermann R, Ochsenbein-Kölble N. Determinants of successful breastfeeding initiation in healthy term singletons: a Swiss university hospital observational study. Journal of perinatal medicine. 2013;41(3):331–9. doi: 10.1515/jpm-2012-0102 23104852

[27] Rasmussen KM, Hilson JA, Kjolhede CL. Obesity may impair lactogenesis II. The Journal of nutrition. 2001;131(11):3009S–11S. doi: 10.1093/jn/131.11.3009S 11694637

[28] Amir LH, Donath S. A systematic review of maternal obesity and breastfeeding intention, initiation and duration. BMC pregnancy and childbirth. 2007;7(1):1–14. doi: 10.1186/1471-2393-7-9 17608952 PMC1937008

[29] Yurtsal ZB, Kocoglu G. The effects of antenatal parental breastfeeding education and counseling on the duration of breastfeeding, and maternal and paternal attachment. Nutrition and Metabolism. 2015;2(4):222–30.

[30] Biks GA, Tariku A, Tessema GA. Effects of antenatal care and institutional delivery on exclusive breastfeeding practice in northwest Ethiopia: a nested case–control study. International breastfeeding journal. 2015;10(1):1–6. 26594231 10.1186/s13006-015-0055-4 PMC4653867

[31] Abebe AM, Wudu Kassaw M, Zemariam AB, Estifanos Shewangashaw N. Coverage, opportunity, and challenges of expanded program on immunization among 12–23-month-old children in woldia town, northeast Ethiopia, 2018. BioMed research international. 2019;2019. doi: 10.1155/2019/5302307 31976321 PMC6955143

